# Cellulose-Based Hydrogels and Aerogels Embedded with Silver Nanoparticles: Preparation and Characterization

**DOI:** 10.3390/gels7030082

**Published:** 2021-07-02

**Authors:** Alexander Vasil’kov, Margarita Rubina, Alexander Naumkin, Mikhail Buzin, Pavel Dorovatovskii, Georgy Peters, Yan Zubavichus

**Affiliations:** 1 A. N. Nesmeyanov Institute of Organoelement Compounds, Russian Academy of Sciences, 28 ul. Vavilova, 119991 Moscow, Russia; alexandervasilkov@yandex.ru (A.V.); naumkin@ineos.ac.ru (A.N.); buzin@ineos.ac.ru (M.B.); 2 National Research Centre “Kurchatov Institute”, 1 pl. Akademika Kurchatova, 123182 Moscow, Russia; dorovatovskiy_PV@nrcki.ru (P.D.); peters_GS@nrcki.ru (G.P.); 3 Federal Research Center Boreskov Institute of Catalysis, Lavrentiev Ave. 5, 630090 Novosibirsk, Russia; yvz@catalysis.ru

**Keywords:** cellulose, silver NPs, bio-aerogel, metal vapor synthesis, organosol, composites, microcrystalline cellulose, aerogel, nanocomposites

## Abstract

The paper presents the preparation and characterization of novel composite materials based on microcrystalline cellulose (MCC) with silver nanoparticles (Ag NPs) in powder and gel forms. We use a promising synthetic conception to form the novel composite biomaterials. At first MCC was modified with colloidal solution of Ag NPs in isopropyl alcohol prepared via metal vapor synthesis. Then Ag-containing MCC powder was used as precursor for further preparation of the gels. The hydrogels were prepared by dissolving pristine MCC and MCC-based composite at low temperatures in aqueous alkali solution and gelation at elevated temperature. To prepare aerogels the drying in supercritical carbon dioxide was implemented. The as-prepared cellulose composites were characterized in terms of morphology, structure, and phase composition. Since many functional properties, including biological activity, in metal-composites are determined by the nature of the metal-to-polymer matrix interaction, the electronic state of the metal was carefully studied. The studied cellulose-based materials containing biologically active Ag NPs may be of interest for use as wound healing or water-purification materials.

## 1. Introduction

According to one of the existing definitions, “an aerogel is a solid material with meso- and macropores with diameters up to a few hundred nanometers and a porosity of more than 95% in which the dispersed phase is a gas” [1]. This class of materials possesses high porosity, low density, and large surface area. Polysaccharide-based aerogels including cellulose-based aerogels are considered in scientific community as a third generation aerogels, so called “bio-aerogels” [2,3], after the silica [4], metal oxide [5], and synthetic polymer-based ones.

The basic principles for preparing cellulose aerogels are the same as for other “bio-aerogels”: the dissolution of biopolymer in appropriate solvent, gelation, exchange of solvents followed by an extraction of liquid phase from gel [3]. To dissolve cellulose different types of solvents are used. Most popular of them are non-derivatizing solvents such as imidazolium-based ionic liquids, phosphoric acid-based solvents, LiCl-based solvents and N-methylmorpholine N-oxide/water [6,7]. The binary system water-sodium hydroxide is an environmentally friendly and cheap solvent for cellulose at temperatures below zero [8]. When cellulose is dissolved in such a solvent, a viscous transparent sol is formed, which at an elevated temperature of 60 °C turns into a physical cellulose II gel with net-like morphology [9].

The step associated with the extraction of the solvent from the pores of the gel is extremely important. Extraction of the liquid phase using supercritical carbon dioxide (sc CO_2_) allows one to preserve the three-dimensional framework of the gel and leads to the production of materials with high specific surface areas [10]. Many bio-aerogels, especially the ones based on polysaccharides such as sodium alginate, pectin, and chitosan, are obtained by drying in sc CO_2_, [11,12,13,14]. Note that sc CO_2_ might also provide sterilization of the treated materials. Indeed, after sc CO_2_ processing, materials are suitable for their further applications in biomedicine, pharmaceuticals, and tissue engineering [15].

Due to its special structural and functional properties, such as high-specific surface area, open pore system, biodegradability and biocompatibility, cellulose hydrogels and aerogels can be used in food, pharmaceutical, cosmetic, environment, and biomedical industrial sectors as carriers of bioactive compounds [16] and drugs [17,18], as a tissue engineering substrate for cultivating organs/tissues/cells [19], as a safety bio-packaging [20] and thermo- [21] and electromagnetic insulation materials [20], and as good adsorbents for water purification [22,23,24] and oil/water separation processes [25]. More examples of the applications of cellulose-based hydrogels and aerogels can be found in recent reviews [26,27,28]. The use of hydrogels and aerogels as containers for functional nanoparticles represents an especially popular trend [29,30].

The inclusion of silver nanoparticles (Ag NPs), which are known to have intrinsic antimicrobial activity [31], into hydrogels and aerogels based on cellulose seems to be a very promising task. Such composite materials are of interest in the field of biomedical practice for the treatment of wounds [32,33], in catalysis as renewable catalysts [34], and as materials for air and water purification for ecology-related applications [35].

One of the effective methods for preparing metal-composites is metal vapor synthesis (MVS). The method is based on the joint condensation of metal vapors with organic reagents in an evacuated reactor, the walls of which are cooled by liquid nitrogen. As a result, colloidal solutions of metal NPs in various organic media-organosols can be prepared. The organosols can be used to modify various types of substrates. MVS is suitable for preparing “pure” metal-composites since no by-products are formed during the synthesis.

In traditional methods of synthesizing silver nanoparticles, environmentally hazardous surfactants and reducing agents are used. In addition, toxic chemicals used to apply nanoparticles to various surfaces containing toxic chemicals that limit their medical applications. Unlike most methods for formation nanoparticles, MVS is completely environmentally friendly and can be easily integrated into various technological cycles. For the composites obtained by MVS, not only their bactericidal and bacteriostatic activities against *E. coli*, *S. aureus*, and *S. enterica* were evaluated, but also toxicological tests were carried out on laboratory mice. It has been shown that such composites used in certain concentration ranges do not exhibit toxicity in the short (3 days) and long (14 days) term in experimental animals [36].

This feature of the method and its versatility as compared to more conventional methods of preparing metal-polymer composites make it particularly promising to produce materials for biomedical purposes. Organosols obtained with MVS have been successfully used to modify biopolymer powders, such as chitosan [37], porous collagen-chitosan scaffold [38,39], bacterial cellulose film [40], and medical bandage [41].

Composite powders obtained with the organosol modification can be further processed into new forms of biomaterials, including hydrogels and aerogels. We have demonstrated that this concept can be successfully applied to obtain hydrogels and aerogels based on chitosan and Ag NPs and Cu NPs [42,43]. To date, the antimicrobial activity of metal-composite biomaterials obtained with MVS against hospital strains of bacteria [41] and excellent fungicidal activity, i.e., counteracting pathogenic microorganisms from dairy cattle feed [43] and fungi infecting potatoes and tomatoes [44] have been demonstrated.

Preparation of new forms of biomaterials from powder metal-polymer composites by the MVS method opens up new routes for obtaining new materials based on the most widespread biopolymer on earth-cellulose. This paper presents a study of nanocomposites based on cellulose in powder and gel (hydro and aerogels) forms with AgNPs.

## 2. Results and Discussion

### 2.1. Preparation, Morphology, and Porous Structure of the Composites

**Figure 1:**
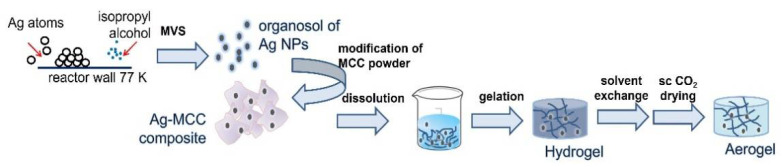
Scheme illustrating the main steps of preparation cellulose-based composites with Ag NPs in powdered and gel form.

**Figure 2:**
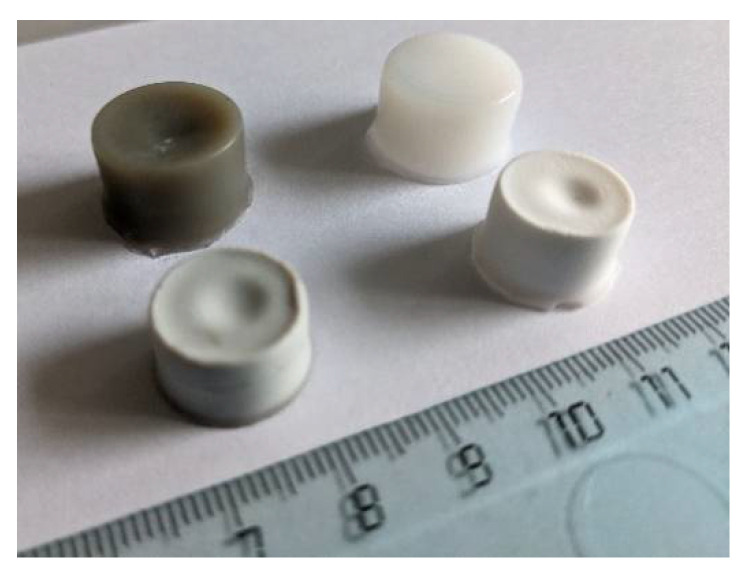
Digital photo of the prepared cellulose hydrogels (top row) and aerogels (bottom row).

The preparation of hydrogels and aerogels based on cellulose and silver nanoparticles includes several stages (Figure 1): preparation of a dispersion of Ag NPs in isopropyl alcohol (organosol) via MVS, modifying powdered MCC using the prepared organosol, and forming a porous 3D gel network from the modified MCC using gelation procedure followed by drying in sc CO_2_. As a result of all the described procedures, cellulose hydrogels and aerogels were obtained, both with and without Ag NPs (see digital photos on Figure 2).

The equilibrium water content in the hydrogels was 91.4 ± 0.2% and 91.5 ± 0.1% for the hydrogel based on MCC and Ag-MCC composite hydrogel, respectively. When changing solvents from water to methanol, no significant shrinkage was observed, while after supercritical drying of the gels, shrinkage was around 20 vol.%. The presence of metal did not affect the shrinkage parameters.

The metal content in the prepared aerogel is close to that of initial metal-containing powder. That means that the multi-step procedure of aerogel preparation does not lead to any significant leaching of the metal, which in turn indicates the strong binding of the metal and the polysaccharide matrix.

Some important characteristics of the prepared aerogels, namely bulk density, porosity, as well as percentage of shrinkage are listed in Table 1.

For calculation of bulk density, the dimensions of the cylindrical form and mass values were used. The porosity (P, %) value was estimated from the bulk ($\rho_{bulk}$) and skeletal density ($\rho_{sk}$) according to equation:

P=(1−ρbulkρsk) × 100%

Total pore volume ($V_{\mathrm{total}}$, cm^3^ g^−1^) of the aerogels was estimated according to equation:

Vtotal =1ρbulk−1ρsk

The value of the skeletal or “true” density of the cellulose was taken from the literature [45] and was approximately 1.5 cm^3^ g^−1^.

**Figure 3:**
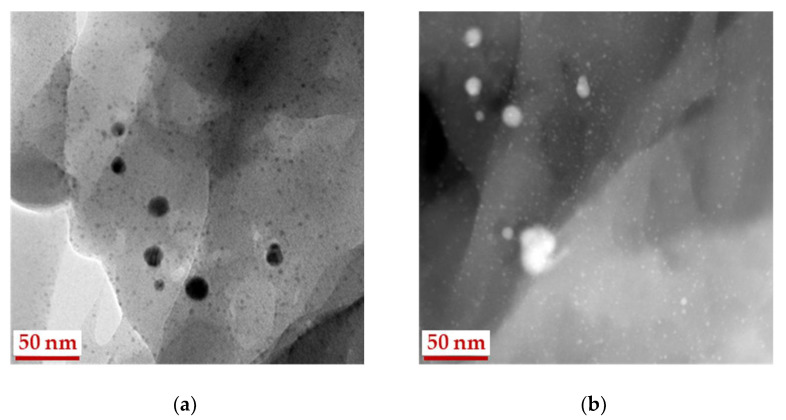
HRTEM images of silver particles in Ag-MCC composite after deposition Ag NPs on microcrystalline cellulose, in bright (**a**) and dark (**b**) fields.

In Figure 3 HRTEM micrographs of silver particles in Ag-MCC composite are presented. The dominant fraction of particles has sizes of 1.5–4.0 nm. There are also larger particles of up to 25 nm in size. Despite the small specific surface area (up to 5 m^2^ g^−1^), MCC, like most biopolymers, has micropores that can participate in stabilization processes. We have previously shown that the presence of micropores and mesopores in the polysaccharide chitosan plays one of the key roles in the stabilization of small metal particles (2–4 nm) [42].

**Figure 4:**
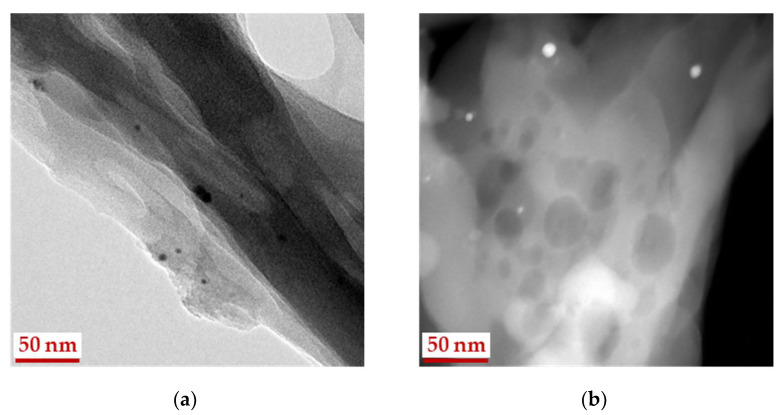
HRTEM images of silver particles in Ag-AC composite aerogel prepared from Ag-MCC, in bright (**a**) and dark (**b**) fields.

Formation of aerogel from powdered MCC is accompanied by a tendency of particles to stick together, forming aggregates. Figure 4 shows images of individual particles with sizes of about 8–10 nm in dark and bright fields for the Ag-AC composite. The aggregates formed from individual particles are also presented in aerogel. The fraction of small metal particles that has been found in MCC fibers is not detected in aerogel. The reason for the observed difference in particle size for matrices is associated both with particle’s aggregation and with a low contrast of the substrate (cellulose fiber) in comparison with the particles.

**Figure 5:**
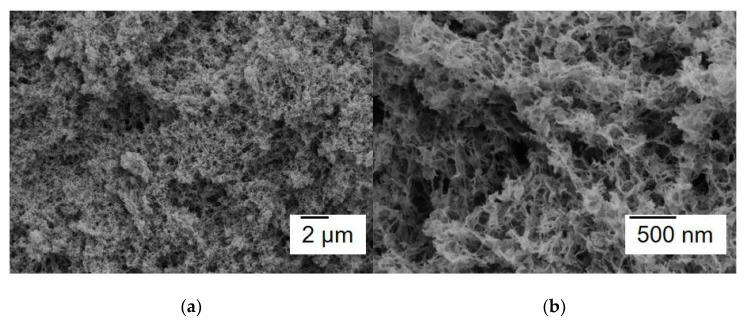
SEM images of the morphology of the prepared composite cellulose aerogel containing Ag NPs: under low (**a**) and high magnification (**b**).

The microstructure of the gels was investigated by SEM. The cross-sections of the samples were examined. In Figure 5, micrographs of the Ag-AC aerogel are presented. As it can be seen, the aerogel has network fibrillar structure forming open pores with sizes of tens nm. Silver NPs are not visualized due to small size.

**Figure 6:**
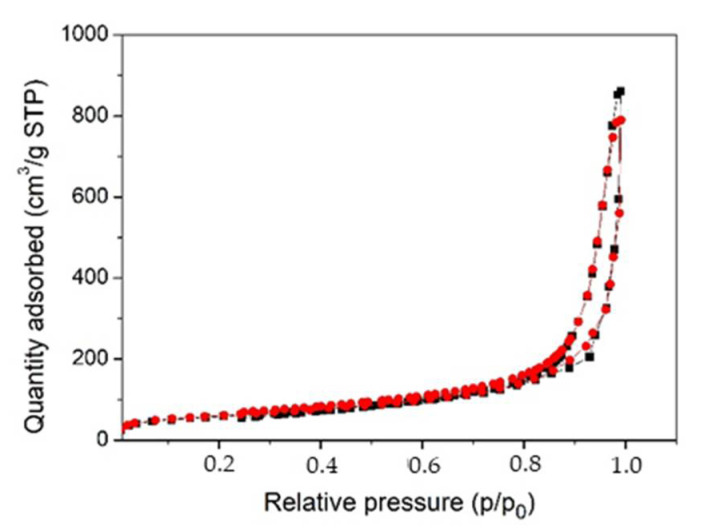
Nitrogen adsorption-desorption isotherms for cellulose-based aerogels: AC (black squares) and Ag-AC (red circles).

The porous structure of the aerogels was studied using the low-temperature nitrogen adsorption method. The aerogels obtained in this work demonstrate the presence of hysteresis loops in the adsorption/desorption isotherms, which is typical of type IV isotherms (Figure 6).

It is known that the form of the hysteresis loop often associated with the form of the pores in adsorbent. According to the accepted classification [45], the isotherms have type H3 loop which means that aerogels are characterized with narrow slit-shaped pores. The specific surface areas (SSAs) of the aerogels determined using the Brunauer, Emmett, and Teller (BET) method are more than 200 m^2^ g^−1^ (see Table 2). A slight increase in SSA is observed for aerogel containing Ag NPs (Ag-AC) in comparison to pristine aerogel (AC). Nonetheless, the absolute precision of SSA measurement by BET is not higher than 5% [46] and it is possible that there is no significant difference between the two values.

**Figure 7:**
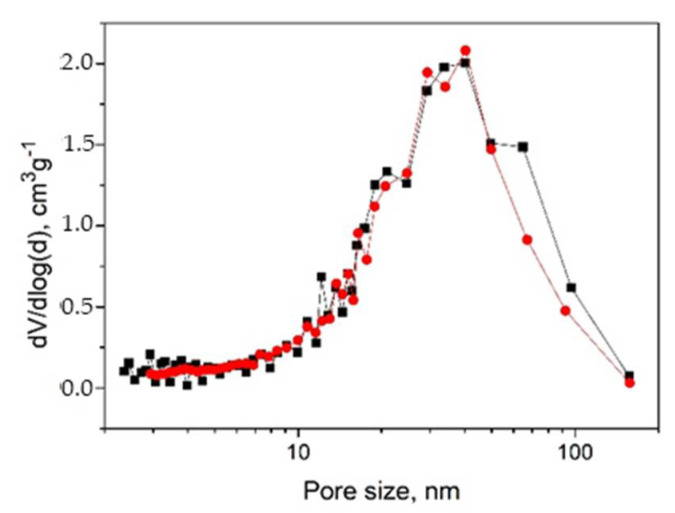
Distribution of total pore volume (differential pore volume dV/dlog(d) vs. pore diameter) determined from BJH model for cellulose-based aerogels: AC (black squares) and Ag-AC (red circles).

The pore size distributions (differential volumes of the pores vs. pore diameter) for aerogels are presented in Figure 7. For the metal-containing and pristine aerogels, the distributions are similar. The general contribution is made by pores with a size of 10 to 70 nm (about 80%), the rest pores with sizes from 2 to 10 nm and larger pores up to 160 nm (the limit for the method).

Total pore volume for cellulose aerogels and mean pore diameter calculated from Barrett–Joyner–Halenda (BJH) model is shown in Table 2. It should be noted that the data obtained from BJH method are not an estimation of the total pore volume in the materials [47]. Close to the true value of the volume is the previously calculated using the values of skeletal and bulk density (see Table 1).

### 2.2. Characterization of the Composites with Synchrotron-Based Techniques

**Figure 8:**
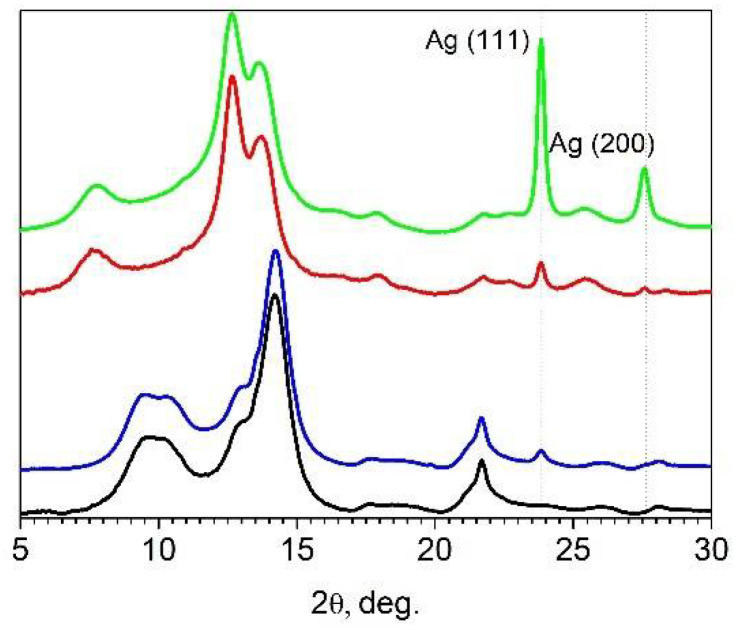
X-ray diffraction patterns (λ = 0.9752 Å) for cellulose samples: MCC (black), AC (red), Ag-MCC (blue) and Ag-AC (green).

X-ray diffraction patterns of pristine and Ag NPs-modified cellulose matrices, including commercial microcrystalline cellulose and cellulose aerogel are shown in Figure 8. The starting commercial microcrystalline cellulose powder is indeed well crystalline. The fraction of amorphous phase is low and does not exceed 15–20%. The crystalline fraction of the sample corresponds to cellulose Iβ polymorph according to positions and relative intensities of diffraction peaks [48,49].

Treatment of cellulose with aqueous alkali solutions causes a transition from native type of cellulose I characterized by parallel packing of chains to mercerized cellulose II polymorph with an antiparallel packing way [50].

The change in the cellulose structure is irreversible and leads to amorphization. For AC obtained as a result of dissolution of MCC in a cooled aqueous solution of alkali, the peak positions and relative intensities of diffraction peaks correspond to cellulose II polymorph [51]. In cellulose samples modified with Ag NPs, additional peaks of the silver fcc phase (111) and (200) are clearly resolvable. The introduction of metal nanoparticles does not significantly affect the crystallinity and crystallite size of the matrices. The size of silver crystallites for Ag-MCC and Ag-AC estimated by the Scherrer equation considering instrumental function is 17 nm.

**Figure 9:**
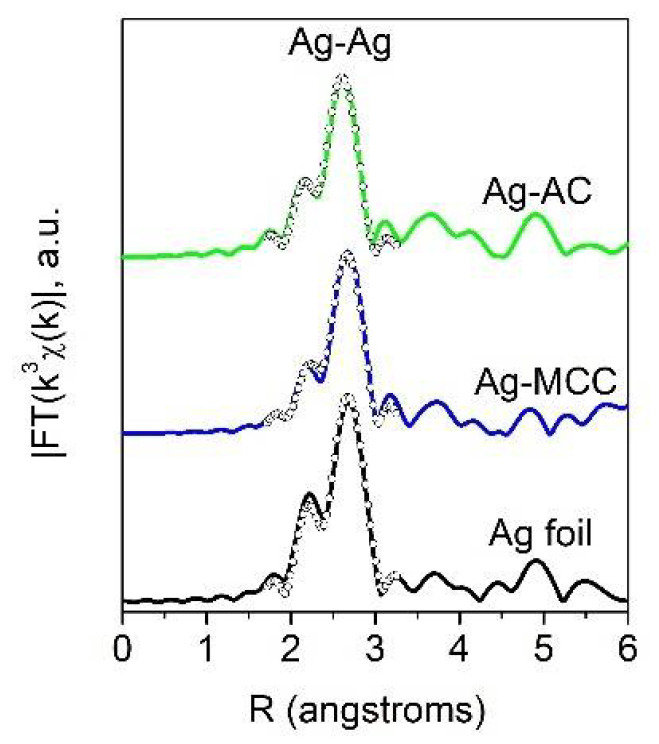
Fourier transforms of Ag K-edge EXAFS spectra for Ag-cellulose composites: experiment (solid lines) and best-fit (open circles).

Structural information on the chemical state and local environment of silver atoms in the cellulose-based metal composites was obtained using the EXAFS method. Fourier transforms of Ag K-edge EXAFS spectra for the four Ag-modified cellulose samples are shown in Figure 9. They are dominated by a single contribution of Ag-Ag interatomic distances very similar to that observed in bulk silver reference. This means that the cellulose nanocomposites contain silver predominantly or even exclusively in the metallic form.

The fraction of oxidized species, including interstitial suboxides AgOx is negligibly small. Some local structure parameters obtained from EXAFS fitting are given in Table 3. As expected for nanoparticles, lowered coordination numbers of the first coordination sphere and shortened Ag-Ag distances with respect to Ag bulk phase are observed in all cases. The shortest Ag-Ag distances are observed for the Ag-AC composite.

**Figure 10:**
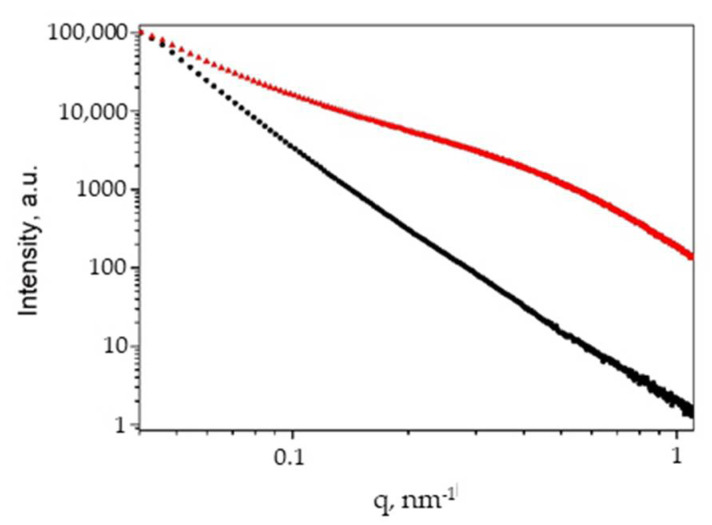
Small-angle X-ray scattering patterns in double logarithmic scale for MCC (black circles) and AC (red triangles).

**Figure 11:**
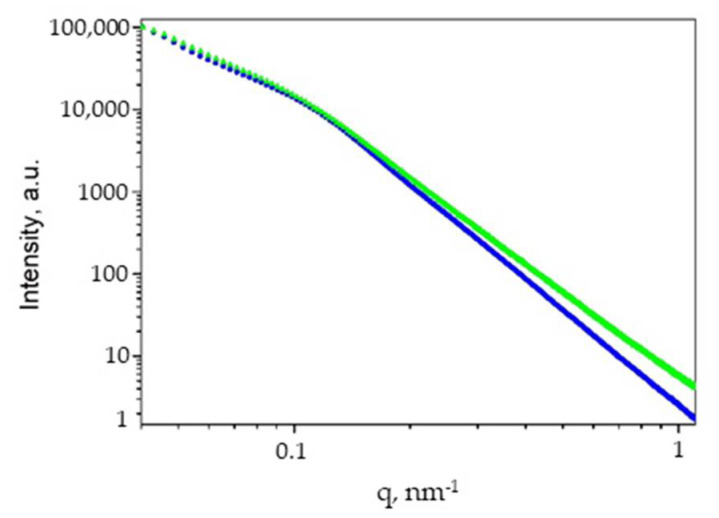
Small-angle X-ray scattering patterns in double logarithmic scale for Ag-MCC (blue triangles) and Ag-AC (green triangles).

Small-angle X-ray scattering patterns of composites and pristine cellulose under study are shown in Figure 10 and Figure 11.

Experimental range of scattered vectors q = 0.04–1.1 nm^−1^ contains information on inhomogeneities of mean density (either regions of increased density or pores) within the samples over the size range 0.9–25 nm. The SAXS curve for pristine microcrystalline cellulose is presented in Figure 8, black curve. In a double logarithmic scale, it looks like a line with a slope α = −3.35. This means that the sample contains no specific structures with characteristic sizes falling into the range indicated above (0.9–25.0 nm) and thus scattering occurs in the high-q Porod’s asymptotic regime [7,52]. The scattering character is governed by rough interfacial boundaries within essentially compact matrix with a surface fractal dimension given by Ds = 6 − α = 2.65. Upon further consideration, we assume that the main scattering centers in AC are cellulose fibrils, which form a porous framework. Thus, analysis of the curves was used to determine the mean filament radii and fractal dimension, as well as to understand the general structure of the solid matrix.

Using SAXS, it was possible to establish the general view of the size distribution of silver particles and to trace the changes in their size when going from MCC to AC. The SAXS curve for the AC (Figure 10, red) reveals a prominent bend that implies that the sample is nanostructured. The experimental curve can be simulated as a superposition of a power-law decrease with α = −2.10 and a Gaussian distribution of spherical scattering centers with a radius R = 2.5 nm and 50% dispersion. The slope of −2.10 is interpreted as mass fractal with respective fractal dimension. Such a fractal can be realized only in the case of abundance of low-dimensional 1D- and 2D-structures like cross-linked networks or polymer cylinders. The morphological parameters of cellulose samples determined by SAXS are summarized in Table 4. The SAXS data are fully consistent with the statement that cellulose aerogels are characterized by highly porous structures. Furthermore, the cellulose aerogel sample is intrinsically nanostructured.

SAXS curves for the metal-filled composites are mainly formed by scattering on Ag NPs due to the high Z-contrast. Two curves reveal a kink in I vs. q decrease implying the existence of a characteristic size describing the morphology (Figure 11). The experimental data were analyzed in a way similar to that described above for the cellulose aerogel, i.e., as a combination of power-law contribution and a Gaussian distribution of spherical scattering centers. However, in this case Ag NPs most probably act as spherical scattering centers, and their aggregates are responsible for the power-law behavior. The corresponding results are listed in Table 4. For composites Ag-MCC and Ag-AC scattering curves are consistent with Ag NPs with radii of 11–13 nm and dispersion of 50%. These SAXS results are in a reasonable agreement with the Ag NPs sizes estimated from XRD line broadening taking into account a rather poor applicability of the Scherrer equation for polydisperse systems with a broad size distribution. The comparative size of silver nanoparticles determined by HRTEM and SAXS, showed their proximity for aerogels and the difference for microcrystalline cellulose, for which HRTEM, in contrast to SAXS, records a significant fraction of small particles. This is due to the fact that HRTEM is a direct method of the measurement, while the particle size determined by SAXS is a function of several parameters and model-dependent.

### 2.3. Surface Analysis of the Composites

XPS analysis was used to study the chemical composition of the cellulose-based nanocomposites. The quantification data based on atomic sensitivity factors are listed in Table 5. Ag/MCC and MCC have the same O/C; ratios (0.6) that demonstrate no-introducing of additional carbon species during the deposition process. Taking into account the formula of the monomer unit of cellulose—(C_6_H_9_O_5_)_n_—the predicted O:C ratio in cellulose should be 0.83 [53]. The original powdered microcrystalline cellulose has a slightly underestimated ratio due to the presence of possible impurities. However, values of 0.7–0.8 are recorded for aerogels, which indicates a relatively pure surface of cellulose in the materials. It seems that the original non-impurities on the surface of MCC are partially recovered. Most likely, the purification of cellulose occurs when alcohol is extracted from the pores of the gel using a supercritical medium, which is known to have excellent penetrating ability [54].

**Figure 12:**
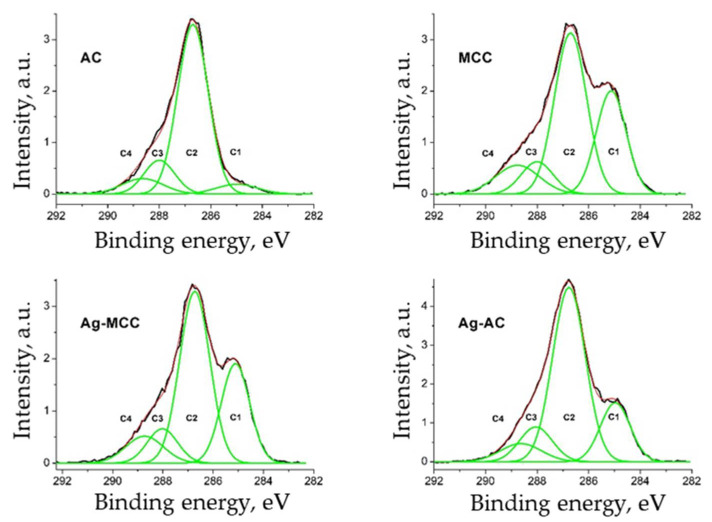
High resolution C 1s core-level spectra fitted with four components for the initial microcrystalline cellulose powder (MCC), cellulose aerogel (AC) as well as corresponding composites with Ag NPs (Ag-MCC and Ag-AC).

Cellulose is a linear polysaccharide consisting of identical fragments of D-glucopyranose linked through β-(1,4)-glycosidic bonds. Thus, cellulose unit contains five carbon atoms bonded to one oxygen and one atom bonded two oxygen atoms; in other words, in the C 1s region two carbon states should be recorded. However, according to numerous publications [55], the C 1s spectra fitted with four peaks. Two additional peaks can be assigned either defects or impurities. Figure 12 shows the C 1s spectra of cellulose samples fitted with four Gaussian peaks, characteristics of which are listed in Table 6.

The C1, C2, C3, and C4 peaks correspond to C-C/C-H, C-O, O-C-O/C = O and O = C–O groups, respectively [56,57,58]. It is worth noting that the C2 and C3 peaks are related to cellulose whereas the C1 and C4 are extrinsically surface adventitious carbons. The presence of adventitious carboxylic group (C4) can be explained with the procedure of preparing microcrystalline cellulose, namely possibly ring opening and oxidation of terminal carbonyl groups [59]. The adventitious carbon atoms C1 should be assigned to strongly bonded organic impurities attending in the raw material of cellulose, lignocellulosic sources [60], or low-molar-mass groups of impurity carbon, and is usually found in all types of cellulose [41,61].

The main differences in the spectra are observed in the regions of low and high binding energies. The relative concentrations of the adventitious carbon atoms (C1 and C4) practically do not change after the deposition of Ag NPs on MCC and decrease for cellulose aerogel (Ag-AC). The transition from MCC to aerogel composites is accompanied by significant decrease of C1 concentration.

**Figure 13:**
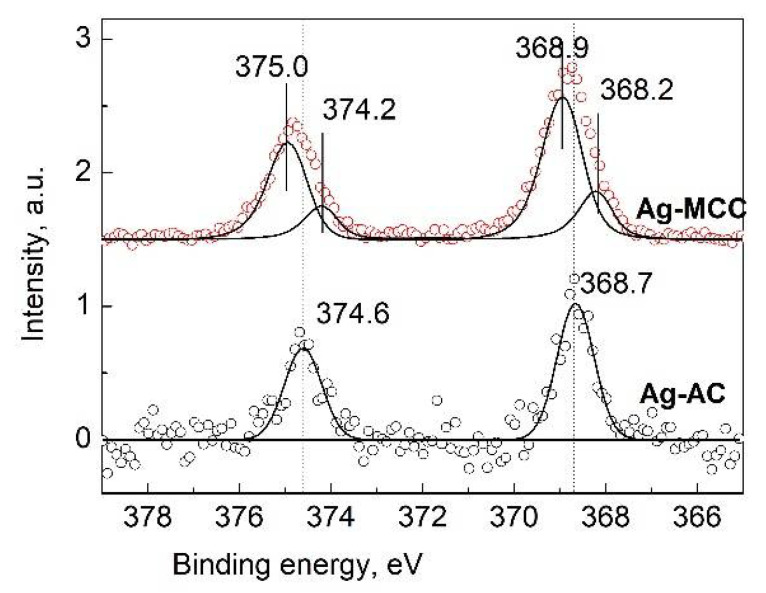
High resolution Ag 3d spectra of microcrystalline cellulose containing Ag NPs (Ag-MCC) and the corresponding aerogel (Ag-AC).

Figure 13 shows the Ag 3d photoelectron spectra of Ag-MCC and Ag-AC composites. As it can be seen, the Ag 3d spectrum of aerogel is characterized with lower signal/noise ratio compared to that of MCC, which is obviously related to the concentration of the metal in the near-surface region. A significant decrease in concentration (about 50 times) on going from MCC to gel is most likely associated with an increase in the specific surface area of the material and good dispersion of metal particles in the bulk.

The Ag 3d spectrum of cellulose aerogel was approximated with two Ag 3d_5/2_ and Ag 3d_3/2_ peaks at 368.7 and 374.6 eV, respectively. The binding energy of the Ag 3d_5/2_ peak of silver in AC is close to unoxidized silver metal (368.3 eV). The positive shift of 0.4 eV is rather associated with size effect. It is well-known that the electronic structure of a metal particle critically depends on its size and as the particle size decreases, the binding-energy shifts increases [62]. At the same time, the charge transfer from metal to support substrate cannot be ruled out [63].

The Ag 3d spectrum of Ag-MCC composite, the precursor of the aerogel shows the noticeable asymmetry and was approximated with two Ag 3d_5/2_-Ag 3d_3/2_ spin-orbit doublets at 368.2–374.2 and 368.9–374.9 eV with ratio 1:3. The peak at 368.2 eV corresponds to bulk unoxidized silver. Taking into account the size effect in photoelectron spectra and the Ag particle size the peak at 368.9 eV may be assigned to Ag^0^ state as well. The greater the binding-energy shift (0.7 eV) to the high-energy region for Ag-MCC in comparison to that of Ag-AC (0.4) is associated with the decreasing of particle size in the matrix. This statement is in good agreement with the electron microscopy data. The micrograph in a dark field (Figure 3, right image) clearly shows 1.5 nm particles uniformly covering the MCC fibers. In the case of an aerogel, such particles are not visible, but particles with a diameter of 8–10 nm are observed (Figure 4).

### 2.4. Thermal Behavior and Stability of the Composites

**Figure 14:**
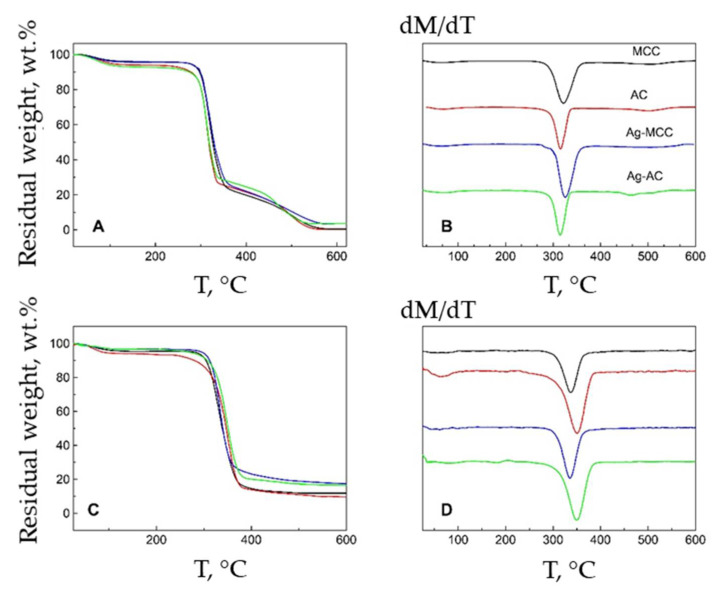
TG and DTG curves for MCC (black), AC (red), Ag-MCC (blue) and Ag-AC (green) in air (**A**,**B**) and argon atmosphere (**C**,**D**) at a heating rate 10 °C/min.

Thermal behavior of powder cellulose and aerogels were analyzed with thermogravimetry in air (Figure 14A,B) and inert atmosphere (Figure 14C,D). The weight loss of cellulose-based composites on the air proceeds in three main stages. At the first stage, ending in the region of 150 °C, the moisture sorbed from the air is removed. The main processes of cellulose decomposition take place in the range of 300–350 °C, with further increase in air temperature, the destructive processes slow down and continue at a significantly lower rate, up to 550 °C. In argon, the last stage of decomposition completely degenerates. Figure 14B,D show the differential thermogravimetric curves. The position of the minimum on the curves indicates the temperature of the maximum rate of decomposition of the sample at the appropriate stage.

It can be seen that the temperature of the maximum decomposition rate in argon for AC (349 °C) is noticeably higher than for MCC (336 °C). It was previously shown that cellulose aerogels have a mesoporous morphology. Thermal conductivity of the material depends on the pore size. With the appearance of pores with diameter less than the average free path of gas molecules (67 nm-air and 64 nm-argon at a pressure of 1 atm), the thermal conductivity is reduced, which affects the higher thermal stability of aerogel in comparison with microcrystalline cellulose. In the air atmosphere, there is an inverse relationship, the cause of which may be explained by the presence in the AC of a developed system of pores and the greater availability of polymer chains to oxidation by air oxygen at elevated temperatures. This opposite behavior of cellulose-based materials upon heating can be explained by a different mechanism of destruction. The effect of silver on the thermal stability of cellulose-based composites was not detected, like what was found in [64,65]. The amount of solid residue in the air of all samples containing metallic silver is the same and amounts to no more than 2%.

## 3. Conclusions

Composite hydrogels and aerogels based on microcrystalline cellulose and Ag NPs were prepared using a combination of MVS, gelation, and drying in sc CO_2_. To obtain gel, microcrystalline cellulose powder modified with sol of Ag NPs prepared via MVS was used as precursor. At each stage of the preparation of the cellulose-based composites, non-toxic reagents, such as isopropanol and sodium hydroxide were used. This approach seems especially promising as it reveals many new possibilities for obtaining various metal-polymer biomaterials.

The as-prepared cellulose composites were characterized in terms of morphology, structure, and phase composition. The dominant fraction of silver NPs in MCC is 1.5–4 nm in size, while in the aerogel particles of 8–10 nm in size are visualized. That is, there is a slight tendency toward aggregation during gel formation. It is revealed that the multi-step procedure of gel preparation does not lead to any significant leaching of the metal, which in turn indicates the strong binding of the metal and the polysaccharide matrix.

Since many functional properties, including biological activity, in metal-composites are determined by the nature of the metal-to-polymer matrix interaction, the electronic state of the metal was carefully studied. It is shown that nanoparticles in the bulk of the material are not oxidized and no specific interaction with the matrix is recorded. Analysis of the surface showed that silver on the surface is also in Ag^0^ state.

The study of the morphology of the gels showed that they are nanostructured, i.e., nano-sized structural elements form a 3D solid network. The cellulose composite aerogel is characterized by an open porous structure with a total porosity of ca. 90%, and a specific surface area of more than 200 m^2^ g^−1^, and a total pore volume of ca. 6 cm^3^ g^−1^ of which ca. 20% is predominantly occupied by mesopores.

Such porous architecture of the biopolymer combined with the presence of antimicrobial NPs might be especially useful in the development of drug carriers with sustained release for pharmaceutics, as well as in the development of new filtration materials with antimicrobial action for water purification.

## 4. Experimental Part Materials and Methods

### 4.1. Materials

Microcrystalline cellulose (MCC) grade Avicel PH-101 with the polymerization degree of 180 and the average particle size of 50μm, denoted in this work as MCC, was purchased from FMC Corp. Methanol and isopropyl alcohol with special purity grade of 99.5%, Ag foils (99.99%) cut on small pieces, and NaOH with purity grade of 98.5% (for analyses) from Acros Organics were used. Prior to the use in the synthesis, the solvent was dried over molecular sieves (4 Å), distilled in an atmosphere of purified Ar and degassed by several consecutive pump-freeze-thaw cycles at 10^−1^ Pa and RT for 1 h.

### 4.2. Preparation of Ag-MCC Composite

The colloidal solution of Ag NPs was prepared with MVS according to the procedure described elsewhere [9]. In a typical experiment 250 mg of Ag (foil, 99.99%) and 120 mL of isopropyl alcohol were taken. Silver was evaporated from a tantalum boat (6 mm × 90 mm). A typical solvent-to-metal molar ratio in the synthesis was 1:500. The resulting colloidal solution was infiltrated into evacuated Schlenk vessels to modify the MCC powder with the mass of 11 g. Then the solvent was removed and modified cellulose Ag-MCC was dried in vacuum at the pressure of 10^−1^ Pa and 40 °C until a constant mass was achieved. Cellulose support was previously degassed in vacuo at 40 °C.

### 4.3. Preparation of Ag-AC Composite

For the preparation of cellulose aerogel with Ag NPs (Ag-AC), previously described procedure was adapted and used [8]. Cellulose powder containing Ag NPs (Ag-MCC) was mixed with water in a beaker and kept for 24 h at 5 °C to allow fiber swelling. Then aqueous solution of NaOH pre-cooled at −6 °C was slowly added to the beaker under vigorous stirring. For preparing 50 g of the solution 2.5 g modified cellulose powder in 30 mL of water and 3.8 g sodium hydroxide in 20 mL of water were taken. Mixing was carried out at −6 °C for 2 h with stirring until a viscous transparent sol was formed. Then the sol was poured into the cylindrical molds (20 mm × 10 mm, 4 g of sol per mold) and left in a thermostat water bath under 60 °C for 2 h to achieve a complete physical gel formation. Then the molds were immersed into a beaker filled with distilled water at room temperature. The gels were thoroughly washed to remove excess of NaOH until neutral pH was reached.

Before drying, water in pores of the hydrogels was replaced with methanol. To a complete solvent exchange, methanol bath was replaced several times for 3 days. At the next stage, the drying in sc CO_2_ was carried out according to the procedure described in [42]. Pristine cellulose aerogel, non-modified with Ag NPs, was prepared as a reference (aerogel type AC).

### 4.4. Characterization

#### 4.4.1. Synchrotron Radiation-Based Techniques

Cellulose-based composites containing Ag NPs were studied by powder X-ray diffraction, small-angle X-ray scattering, and X-ray absorption spectroscopy at the Kurchatov synchrotron radiation source (NRC “Kurchatov Institute”, Moscow, Russia). Powder X-ray diffraction patterns were measured at the BELOK beamline [66] in the transmission mode using a Rayonix SX-165 detector at a wavelength λ = 0.9752 Å and sample-to-detector distance 100 mm. Small-angle X-ray scattering patterns were measured at the DICSI beamline [67] using a Dectris Pilatus 1M detector at a wavelength of λ = 1.6200 Å and sample-to-detector distance 2400 mm. Integration of 2D scattering patterns was performed using the Fit2D code [68]. The size of Ag nanoparticles was assessed from diffraction line broadening in XRD using the Scherrer equation and from SAXS curves using the SAXS Fit code [69]. Ag K-edge EXAFS spectra were acquired at the Structural Materials Science beamline [70] in the fluorescence yield mode using a Si avalanche photodiode. Spectra for the Ag foil reference were measured in the transmission mode using two Ar-filled ionization chambers. Experimental data processing and analysis were performed using the IFEFFIT software package [71].

#### 4.4.2. X-ray Photoelectron Spectroscopy (XPS)

X-ray photoelectron spectra were acquired with a ThetaProbe (Thermo Fisher Scientific, Paisley, UK) spectrometer using monochromatized Al Kα (1486.6 eV) radiation. Survey and high-resolution spectra of appropriate core levels were recorded with step sizes of 1 and 0.1 eV, respectively. The base pressure in the analytical UHV chamber of the spectrometer during measurements did not exceed ~5 × 10^−7^ Pa. The energy scale of the spectrometer was calibrated to provide the following values for reference samples (i.e., metal surfaces freshly cleaned by ion bombardment): Au 4f_7/2_—84.00 eV, Cu 2p_3/2_—932.70 eV, Ag 3d_5/2_—368.30 eV [72]. The surface charging was taken into account in the following way: the C-OH state isolated in the C 1s spectrum of the support and the precursor was assigned a binding energy of 286.73 eV [73].

#### 4.4.3. Porosity, Microstructure, and Morphology

Nitrogen adsorption–desorption isotherms were measured on a Quantochrome Nova Station A at 77 K. Before the measurements, the aerogels were degassed at 60 °C for 6 h. The surface area was calculated from isotherm of adsorption in the relative pressure p/p0 range of ca. 0.035–0.14. Microstructure of the drying gels was studied using scanning electron microscope SEM-FEG Zeiss Supra 40. TEM images were performed with a transmission electron microscope JEOL JEM 2100F/UHR operating at 200 kV.

#### 4.4.4. Thermal Stability

Thermogravimetric analysis (TGA) and dynamic thermogravimetric analysis (DTA) were carried out by DerivatographC (MOM, Budapest, Hungary) at a scan rate of 10°/min from RT to 800 °C under air atmosphere and in argon, respectively.

## Figures and Tables

**Table 1:** Main characteristics of the prepared cellulose aerogels. The error for calculated values lies in the range of 5–10%.

Sample	Volume Shrinkage, %	Bulk Density, cm^3^ g^−1^	Total Pore Volume, cm^3^ g^−1^	Porosity, %
AC (aerogel from pristine MCC)	19	0.15	6.00	90
Ag-AC (aerogel from MCC modified with Ag NPs)	19	0.16	5.58	89

**Table 2:** Textural characteristics of the cellulose aerogels.

Sample	SSA, m^2^g^−1^	BJH Cumulative Pore Volume, cm^−3^g^−1^	BJH Mean Pore Dimeter, nm
AC (aerogel from pristine MCC)	205	1.3	23
Ag-AC (aerogel from MCC modified with Ag NPs)	212	1.2	26

**Table 3:** Parameters of local environment of silver atoms in Ag-cellulose composites according to EXAFS data.

Sample	N	R, Å	σ^2^, Å^2^	ΔE, eV	R_f_
Ag foil	12	2.881	0.0096	3.0	0.010
Ag-MCC	9.9	2.867	0.0096	2.4	0.009
Ag-AC	9.8	2.844	0.0096	−1.7	0.005

Fitting ranges: R = 1.9–3.0 Å, k = 2.0–12.0 Å^−1^.

**Table 4:** Morphological parameters of Ag-cellulose composites from SAXS data.

Sample	Power-Law Slope, α	R, nm	Dispersion, %
MCC	3.35	-	-
AC	2.10	2.5	50
Ag-MCC	3.45	11.0	50
Ag-AC;	3.10	10.8	50

**Table 5:** Elemental composition of the cellulose-based composites.

Sample	Concentration, at.%			
	C	O	Ag	O/C
MCC	63.5	36.5	-	0.6
Ag-MCC	63.1	36.4	0.5	0.6
AC	54.8	45.2	-	0.8
Ag-AC	58.7	41.2	0.01	0.7

**Table 6:** Characteristics of Gaussian peaks in the C 1s spectra.

Sample	Carbon Type	Binding Energies, eV	Peak Width, eV	Relative Intensity
MCC	C1	285.14	1.16	0.29
	C2	286.73	1.24	0.50
	C3	288.02	1.24	0.10
	C4	288.96	1.69	0.12
Ag-MCC	C1	285.11	1.13	0.28
	C2	286.73	1.20	0.52
	C3	288.03	1.20	0.10
	C4	288.95	1.20	0.10
AC	C1	285.01	1.60	0.06
	C2	286.73	1.19	0.71
	C3	288.02	1.19	0.14
	C4	288.65	1.59	0.09
Ag-AC	C1	284.94	1.15	0.19
	C2	286.73	1.27	0.61
	C3	288.02	1.27	0.12
	C4	288.63	1.69	0.08
